# Evidence Based Dentistry among Dentists in Low and Middle Income Countries: A Systematic Review

**DOI:** 10.24248/eahrj.v5i2.662

**Published:** 2021-11-15

**Authors:** Irene Kida Minja, Edda Tandi Lwoga

**Affiliations:** a Department of Restorative Dentistry, School of Dentistry, Muhimbili University of Health and Allied Sciences – Tanzania; b Department of Mathematics and ICT, College of Business Education – Tanzania

## Abstract

Purpose of this systematic review was to bring together studies of evidence-based practice among dentists in low- and middle-income countries, where its use has been reported to be limited. The protocol was registered in PROSPERO. Methodology: We searched the evidence (in English only) from medical databases including PubMed, EBSCO, The Cochrane Library, CINAHL, ScienceDirect, HINARI summon, and SCOPUS and Web of Science via Research4Life, grey literature, hand search from relevant articles, and augmented results on Google scholar. Published reports were retrieved from relevant websites and organizations. Studies included those that looked at key factors that facilitate or hinder Evidence Based Dentistry (EBD), as well as outcomes in terms of: knowledge, attitudes and skills of EB practice among dentists; and the methodology used and their relevance in future EBD strategies. Main focus was on dentists, as practitioners and faculty members. Studies on students and non-dental personnel were excluded. Findings: A total of 4568 records were retrieved and five potentially relevant articles were selected after title/abstract screening. Two articles were excluded after full text screening, and therefore Three papers were included in this review. The studies report limited knowledge, unsatisfactory attitude towards EBD and lowpractice of EBD and use of scientific evidence databases. None of the studies reported implementation of EBD nor evaluation thereof. The main barriers that constrained application of EBD ranged from lack of interest to infrastructural limitations. Originality: The current review showed that there is a need to strategised implementation of EBD in this region.

## BACKGROUND

Globally, there is improvement in accessibility to internet and search engines, leading to an ‘explosion of information’ which is made available widely to both professionals and non-professionals (consumers). This makes the situation complex when it comes to selecting sound research evidence for optimal care, hence, the concept of evidence-based medicine (EBM) was introduced. EBM is defined as a “conscientious, explicit and judicious use of current best evidence in making decisions about the care of individual patients”.^1^ This process integrates individual clinical expertise with the best available external clinical evidence from systematic research^1,2^ It entails developments that take place globally from research activities, should be used as evidence in making decision while managing patients. It is recognised that healthcare is patient specific, constantly changing and involves uncertainties and probabilities.^2,3^ This process is applicable to all health fields, and dentistry included, which is termed as evidence-based dentistry (EBD).

The American Dental Association defines the evidence based dentistry (EBD) as “an approach to oral healthcare that requires the judicious integration of systematic assessments of clinically relevant scientific evidence, relating to the patient's oral and medical condition and history, with the dentist's clinical expertise and the patient's treatment needs and preferences”^4^ (http://www.ada.org/en/about-the-ada/ada-positions-policies-and-statements/policy-on-evidence-based-dentistry).

EBD refers to a broad set of three distinct skills: 1) the building of skills in students and dentists in assessing literature to directly allow them to knowledgeably read, interpret and assess clinically-relevant *original research articles*, i.e. what is often referred to as ‘primary sources of information’ or ‘primary literature articles'; 2) skills to directly allow them to knowledgeably read, interpret and assess clinically-relevant *literature review articles.* This might be either traditional literature review articles or systematic review articles, the latter either with or without meta-analysis (sometimes referred to as *summary articles* or the *summative literature)*; and, 3) the building of Problem/population, Intervention, Comparison group, and Outcome (PICO) skills in students and dentists to generate an answerable question^5^. Ultimately, this will build on how they can incorporate the findings of either *original research articles* or *literature review articles* into their ‘evidence-based dentistry’ decision-making process to provide-‘evidence-based’ care for their patients. Hence, the basis for clinical decision in EBD, involves a triad of best available evidence, together with practitioners' expertise and patient characteristics or preferences.

Utilisation of evidence in patient care has a number of benefits including but not limited to support practitioner's decision-making process as well as enhance trust in treatment by the community.^6,7^ EBD is useful when it comes to assisting in cost containment in health care and positively impacting on patient treatment outcome. EBD also makes practitioners more accountable in their practice; and allows incorporation of clinical research into practice. EBD, when included as an integral part in patient dental care, dental training and research was seen to improve skills and expertise and treatment outcome.

Efforts have been done to integrate evidence-based practice (EBP) into clinical practice worldwide. These efforts have been evaluated across different health fields including general physicians, nurses, occupational therapy, physical therapists, western herbal medicine providers, dentists and the like. Strategies utilized to incorporate EBD included: promoting online knowledge transfer on rapid access to information on EBD among dentists; inclusion of summaries of systematic reviews and recommended treatment and guidelines; and in dental education^5^. Due to the complexity of dental care, use of evidence in dental practice has been reported to be limited. The latter is reported to be due to scarcity of literature on high evidence research such as Randomised Control Trials (RCT)^8^. Models that specifically target dental profession are important due to the level of control a patient has concerning how, when and if it is necessary to treat dental problems in terms of personal desires and insurance benefits^8^. Building a research culture during undergraduate training is one of the basic strategies to inculcate utilisation of research evidence in patient care. In a study that assessed dental and medical students in Saudi Arabia, reported deficient knowledge and attitudes towards EBP and recommended changes that will enhance implementation of EBP.^9^ Studies have revealed marked improvement in students' knowledge, attitudes ands kills to practice EBP following EBP training. This has been observed among nurses, medical, as well as dental students.^10–12^

Most decision making on treating patients in most lowand middle-income countries (LMICs), has been based on what has taken place during normal undergraduate or postgraduate training. Hay and colleaguesalso reported that, most physicians rarely used scientific evidence, but rely on their own or colleagues' experience in patient care.^13^ On the same note, few studies done in LMICs on EBP showed limited use of scholarly electronic journals and the use of non-scholarly information (such as Google) was high.^14–16^ Due to the fact that, developments in dentistry do occur significantly, having good understanding andknowledge on acquiring accurate information is one of the key components that will assist in translating research evidence into clinical practice/community decision making.

There is a lot of primary research regarding EBD among dentists when it comes to their knowledge, practice and skills; as well as implementation strategies. The reported research has mostly been conducted in the upper middle-and high-income countries. The objective of this review, therefore, was to bring together adequate studies that assess the implementation of EBP among dentists in low and middle and income countries. We looked at the methodology used if they are adequate; and also, the key factors that facilitate or hinder EBD, as well as outcomes (knowledge, attitudes and skills) of EB practice among dentists, to assist in recommendation of future strategies.

## METHODOLOGY

The protocol for the current study was registered with PROSPERO databases in 2018, ID CRD42018090216.

### Criteria for Considering Studies for this Review Types of Studies

We included all cross-sectional survey studies, qualitative studies, case-control studies, randomised controlled trials, quasi-experimental studies, and cohort studies with or without comparison groups.

### Participants/Population

We included studies that had focused on dentists, as practitioners and faculty members. We excluded dental students as well as other studies that had focused on both dentists and other medical practitioners such as medical doctors.

### Intervention

We included studies that had the application of Evidence Based Dentistry (EBD) among dentists in terms of having knowledge and skills on acquiring accurate information and utilization of scientific evidence in patient care.

### Main Outcome

The study assessed the improved levels of knowledge, skills, attitudes and practice of evidence-based dentistry among dentists from the low- and middle-income countries. The study compared the change of knowledge, skills and attitude and before and after the implementation of EBD. Additional outcomes included the following: Change of behavior towards EBD practice among dentists, formulation of guidelines, improved search strategies and discussions in meetings after implementation of EBD

### Search Methods for Identification of Studies

We searched evidence from medical databasesin November, 2018, and we updated the literature between October and January, 2020. These databases includedPubMed, EBSCO, The Cochrane Library, CINAHL, ScienceDirect, HINARI summon, and SCOPUS and Web of Science via Research4LIfe. Moreover, we conducted a grey literature search from respective databases and conducted a hand search from relevant articles in the subject matter. We also searched Google scholar to augment results from other databases. Relevant literature and published reports were retrieved from other websites and organizations. We searched literature published in English only and without restricting time period. The search terms that were used for each database are attached in Appendix 1 (http://tinyurl.com/EBDappendix1).

### Screening

Two review authors conducted the search and screened the research studies, by titles and abstracts for inclusion. Further, the authors conducted the full-text screening. Agreement on their inclusionwas reached via consensus. Rayyan software was used to conduct the title/abstract screening, while CADIMA was used for full text screening. An experienced researcher was consulted in case of the discrepancies during data screening.

### Data Extraction

Data extraction was carried out under the guidance of the PRISMA checklist17. Two review authors independently extracted data from the included studies using a standardized data extraction form that was created in CADIMA. Afterwards, the data compared. An experienced researcher was consulted in case of the discrepancies during data extraction.

### Risk of Bias Assessment

The risk of bias of the included studies was assessed by using the scale for quantitative and qualitative studies developed by Kmet and co-workers18. The tool for the quantitative studies has 14 items, which can be scored based on the degree to which the specific criteria were met (“yes” = 2, “partial” = 1, “no” = 0). The items that were not applicable to a particular study were scored as “N/A”18. Thus, they were not included from the summary score. Therefore, the “summary score for each paper was calculated by summing the total score obtained across the 14 items and dividing by 28 of the total possible score”18. The tool for qualitative studies had ten items, and the scores can be calculated in a similar fashion as for quantitative studies. The ‘not applicable’ option is not allowed for quantitative studies. Therefore, the “summary score for each paper was calculated by summing the total score obtained across the ten items and dividing by 20 of the total possible score”18. Two raters (authors) performed independently the risk of bias and then resolved differences.

### Data Analysis

Structured narrative review of the studies is presented in this review. We used Excel to code and categorize data for qualitative data analysis.

## FINDINGS

### Search Results

**FIGURE 1:**
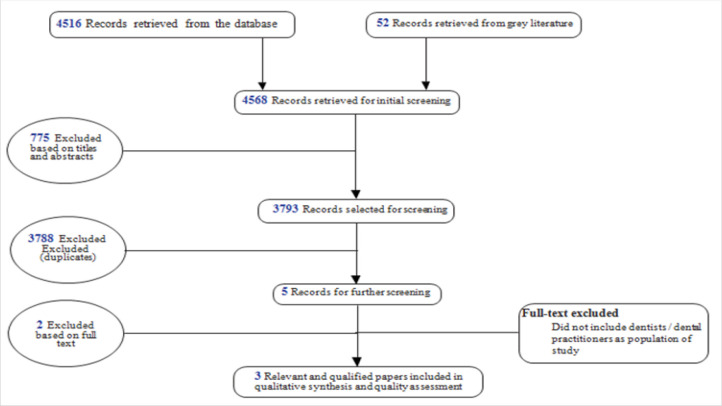
Flow chart diagram: Publications screening process to get relevant papers that qualified for data synthesis and quality assessment

The search retrieved 4568 records, and 775 duplicated records were excluded (see Figure 1). About 3793 were selected for further screening, and 3788 were excluded following title and abstract screening. About five potentially relevant articles were selected, and two articles were excluded following full text screening based on the pre-specified inclusion and exclusion criteria. About three papers were subjected for quality assessment, and they were further included in the qualitative analysis.

### Study Quality, the Risk of Bias, and Quality of Evidence

The risk of bias was further assessed for the two quantitative studies that were selected. The overall scores (Table 1) assigned by both reviewers was 0.9. Both reviewers allocated the same overall score to the two studies. The risk of bias was further assessed for the one qualitative study that was selected. The overall scores (See Table 2) assigned by both reviewers was 0.5. Both reviewers assigned the same overall score to the study.

**TABLE 1:** Risk of Bias Results for Quantitative Studies

Criteria	Adeoye, 2008	Nzabonimana et al., 2019
1. Question/objective sufficiently described?	2	2
2. Study design evident and appropriate?	2	2
3. Method of subject/comparison group selection or source of information/input variables described and appropriate?	2	2
4. Subject (and comparison group, if applicable) characteristics sufficiently described?	2	2
5. If interventional and random allocation was possible, was it described?	N/A	N/A
6. If interventional and blinding of investigators was possible, was it reported?	N/A	N/A
7. If interventional and blinding of subjects was possible, was it reported?	N/A	N/A
8. Outcome and (if applicable) exposure measure(s) well defined and robust to measurement/misclassification bias? Means of assessment reported?	2	2
9. Sample size appropriate?	2	2
10. Analytic methods described/justified and appropriate?	1	1
11. Some estimate of variance is reported for the main results?	1	1
12. Controlled for confounding?	N/A	N/A
13. Results reported in sufficient detail?	2	2
14. Conclusions supported by the results?	2	2
**Total**	**18/20=0.9**	**18/20=0.9**

Key: 2 = Yes; 1 = Partial; 0 = No

**TABLE 2:** Risk of Bias Results for Qualitative Study

Criteria	Adeniyi & Adeyemo (2010)
1. Question/objective sufficiently described?	2
2. Study design evident and appropriate?	2
3. Context for the study clear?	2
4. Connection to a theoretical framework/wider body of knowledge?	1
5. Sampling strategy described, relevant and justified?	0
6. Data collection methods clearly described 1 and systematic?	1
7. Data analysis clearly described and systematic?	0
8. Use of verification procedure(s) to establish credibility?	0
9. Conclusions supported by the results?	2
10. Refiexivity of the account?	1
**Total**	**11/20=0.55**

Key: 2 = Yes; 1 = Partial; 0 = No

### Methodological Issues of the Included Studies

There was a total of three articles that fulfilled the criteria and were qualified in qualitative synthesis and quality assessment.^19–21^ Two studies were conducted in Nigeria and one in Rwanda.^19–21^ Two of these studies involved assessment of knowledge, attitude and practice of dental practitioners and faculty on EBD.^19,20^ The third article aimed to determine the level of knowledge, attitude, and practice of oral health care providers toward the use of online medical databases for clinical decision-making processes.^21^ None of the studies reported presence of EBD implementation strategies nor evaluation thereof. The first article aimed to assess the state of EBD in the four fully accredited dental schools in Nigeria as an example of a developing economy.^20^

This study involved, firstly, literature search on EBD that included six articles. Secondly, faculty members, dental specialists and resident doctors were interviewed over the phone. Among the six articles retrieved from literature search, three of themreported on steps by step procedures to follow on how to conduct EBD practice; while the other two reported on application of EBD for different dental procedures; the last one was a letter to the editor.^22–27^ The second reviewed article conducted questionnaire survey among dentists registered with medical and dental council (N=114) in Nigeria^19^. While the third article involved oral health care providers (N=201) who are registered either with the Rwanda Allied Health Professional Council (RAHPC) or Rwanda Medical and Dental Council (RMDC)^21^. The latter also utilized self-administered questionnaires. See Appendix 2 for more details (http://tinyurl.com/EBDappendixII).

The studies were ethically approved by their governing bodies and permission to conduct studies were reported as University of Rwanda College of Medicine and Health Sciences (CMHS) Institutional Review Board and permission to obtain contact details from RAHPC and RMDC; and the Senate Research University of Western Cape^19,21^.

### Awareness and Knowledge on EBD

All studies report that knowledge and awareness of EBD to be below averageor of low level.^20.21^ Similarly, on assessment of knowledge of different online databases, Adeoye reported thatthat slightly over 50% were not aware of the Cochrane collaboration while about 20% had minimal knowledge of the same^19^. In addition, whereas systematic review could be defined by 42% of participants, 25.4% of them were not aware of the systematic review terminology. Critical appraisal terminology was understood by about 50%, but more than a third of them were not aware of it^19^. Less than one third of the respondents could choose correct definitions of EBD related terms, including evidence-based practice, critical appraisal and systematic review (30.7%, 31.6% and 21.1%, respectively). Among those who asserted to have knowledge on the three terminologies of EBP, systematic review and critical appraisal, only 32.5%, 40.4% and about half (54.4%) had truly answered correctly^19^. On the same note, Nzabonimana et al., reported that participants had low level of awareness of useful databases for clinical decision making such as PubMed (41%), Drug. com (29%), Medscape (16%), and MedlinePlus (14%).^21^

### Attitude towards of EBD

The reviewed studies indicated that poor attitude towards EBD was observed in most primary studies. Contrary to this, Adeoyereported that almost all (97.4%) the participating dental practitioners were interested in getting more informed on EBD with 42.1% desiring short courses as way to get the information.^19^ Additionally, the latter study stated that 46.9% of those with awareness on EBD concept feltthat it was important while 17.7% felt the EBD concept was not important.^19^ Similarly, Nzabonimana et al., reported that positive attitude towards the use of online medical databases and resources is important to support clinical decision^21^. Furthermore, the oral care providers in this study, also believed availability of online resources is beneficial for patient care.

### EBD Practice

The results indicate that there was low level of practicing EBD among dental professionals in the reviewed studies. EBD was not practiced by most dental practitioners, with majority utilizing clinical experience and expertise as the main basis for decision making during patient care at their centers.^20^ Nzabonimana and co-workersin their assessment of utilization of online resources for clinical decision making, reported that slightly less than half (49.7%) of the oral health care practitioners agreed that use of online databases was useful for clinical decision making while 20.4% disagreed and 29% where neutral.^21^

### Basis for decision making in patients care and information sources consulted

The results indicate that electronic resources and person to person communication as the main sources of information for dental professional'spatient care services. For instance, Nzabonimana found that most dental professional relied on Google (81%) and 60% used YouTube videos for professional needs.^21^ Adeoye reported that, majority of the dentists (68.4%) consulted either friends/colleagues opinion; 18.4% used textbooks and 7.9% electronic database when uncertain about choices of treatment^19^. The study among Rwandese oral health care provides reported information sought for was about clinical procedures (34.8%), drug prescription (24.8%) and drug interaction (5%).^21^

### Implementation Strategies of Evidence-Based Dentistry

None of the studies retrieved for this review reported structured implementation strategies on evidence-based dentistry by institutions or hospitals for students, postgraduates, faculty or dental/oral clinicians. All studies reported on knowledge, attitudes and practice of EBD. Therefore, evaluation of implementation was not applicable.

### Behavior Changes

Adeoye reported that majority of the participants (82.3%) with awareness on EBD agreed to have changed their behavior to use the EB concept during practice^19^. The change was attributed to reading articles on EBD. The change in practice was also attributed to the quality of the journal articles read, the types of journals as well as the authors. Very few respondents (N=10) in the study reported to have participated in EBP courses.

### Established EBD Activities

None of the articles reviewed reported any formal and structured EBD training for clinicians or dental students.

### Barriers for EBD Practice

Two of the studies reported barriers perceived by dental practitioners towards practicing EBD. Adeniyi and colleaguereported five barriers as outlined here: firstly, deficient practitioner interest; secondly, in dental health care, conventional model was more convenient, beneficial and effective than the EB model; thirdly, for most dental health conditions, reliable and high-level evidence is scarce, and that the retrievable evidence may not be applicable in local settings as they are from dissimilar cultures^20^. Fourthly, challenges in infrastructure such as unreliable electricity power supplies, limited access to web-based subscription medical databases, computers and internet services; and lastly, scarcity of mentors who are passionate about EBD. Additionally, Adeoyereported the most mentioned barriers being limited knowledge and awareness of EBP, inadequate finances, as well as equipment and materials^19^. Not having enough time for EBD practice was also cited as an obstacle due to high workload. Also stated was the fear related to slow rate of acceptance by dentists and limited opportunities for training EBP.

## DISCUSSION

### Methodological Issues

In this review, there were three articles that fitted the criteria for inclusion into this review, showing the scarcity of EBD research in LMICs. Similarly, the current evidence on implementation strategies of EBD in LMICs is lacking. We expected to carryout meta-analysis and therefore to deploy meta-regression analysis by age, geographical location as well as other demographic characteristics. This analysis could not be performed due to a limited number of quantitative studies that are comparable. Despite the minimal number of articles included, systematic review can assist to identify that as a gap and form basis to suggest further work on the area of interest.

Methodologically, the included studies mainly focused on the cross-sectional survey methods and qualitative interviews. Further, it is worth noting that the risk of bias assessment for qualitative studies was moderate, while the rating for the quantitative studies was above 0.9 percent. These findings indicate a good quality of the applied methods in the included studies. However, since the included studies only applied cross-sectional surveys, and minimal use of qualitative methods, there is a need for a more rigorous mixed method studies that will combine both quantitative and qualitative studies. Qualitative studies will assist to understand the context of EBD, while quantitative studies e.g., Randomized Controlled Trials (RCTs) will enable to ascertain the extent of the application of EBD, and areas for improvement.

### Knowledge, attitude and practice of EBD

The reviewed studies report low and below average knowledge and awareness of EBD and low use of EB databases.^19–21^ Significant number of dentists with limited levels of knowledge on EBD has been reported elsewhere in countries like Iran and Pakistan^28,29^. Studies reveal that having knowledge on issues pertaining to EBD, increases chances on uptake of EBD in patient care decision making^12^. Currently, access to internet has increased globally, this is true also for the LMICs. The improved access to information, necessitates the need to strategize implementation of EBD, particularly, to build capacity in utilization of appropriate online peer reviewed medical resourcesand tools for clinical decision making. Training in the form of continuing educational programs to clinicians and faculty; and incorporation of EBD modules into degree/certificate programs has been recommended.^28,29^

A positive attitude is essential for a change to occur towards a desired outcome. The current reviewed studies showed variation inreported attitudes of the dentists towards EBD. The dentists in the study byAdeniyi and colleague, reported negative attitudes towards utilization of EBD, unlike the other two studies that showed positive attitudes towards EBD^19–21^. The negative attitudes stated were mostly related to lack of interest as well as unfavorableconditions for EBD practice, is a common phenomenon in most LMICs. This calls for interventions geared towards not only improved knowledge and attitudes but also making available the required tools to make EBD practical. The latter efforts are also applicable and may bring more effective outcomes, in situations where attitudes are positive among the dentists^19,21^.

Practice of EBD and use of scientific evidence databases was low among the dentist in the current review. Moreover, even though a substantial number of dentists reporting EBD practice in the current review, a large number could not correctly respond to the EBD knowledge questions.^19^ As reported in a previous study in Kuwait, despite the high percentage of dentists reporting the use of EBD, fewer than fifty percent had reasonable understanding of fundamentals related to EBD.^30^ Majority of the dentists rely on experienceand obtaining information from colleagues or textbooks and non-peer reviewed information, which are convenient to them, but are considered not to have sufficient evidence for decision making. The study found that electronic resources and person to person communication as the main sources of information for dental professionals' patient care services. On electronic sources, the findings further showed that dental professionals relied on the Google (81%) and YouTube videos (60%) for professional needs. The high use of Google search has also been reported in other studies^16^. This finding indicates that the use of e-resources is discouraging as one would have expected probably due to lack of awareness and skills on how to use e-resources. Although Google is a good resource for retrieving large amount of results for dental professionals, concerns have been raised about how reliable is the information that it retrieves.^16^

### Implementation of EBD

The lack of studies, in this review, reporting implementation strategies for EBD in LMICs justifies why there was low or below average levels of knowledge on EBD. On the same note, EBD interventions, could improve dentists' attitudes towards the use of evidence in decision making and hence practice EBD. This underscores the need for EBD interventions among dentists in the region.

### Perceived barriers to practice EBD

There was a high number of literaturesthat assessed factors affecting uptake of EBP with more focus on medical profession. Despite the similarities in identified factors associated with EBP in other professions with those for dental profession, there is considerable differences in medical and dental practice due to differences in funding and organizational structures^31^. Accessibility to dental literatures, insurance coverage to name the few, require plans that will be based on barriers and promoters specifically for EBD^31,32^

As reported a systematic review previously, shortage of time and financial constraints featuring as barrier to EBD practice^33^

In our review similar barriers were noted, and in addition, cultural issues and non-supportive infrastructural were mentioned, requiring cultural and societal sensitive evidence to be obtained for effective implementation of evidence-based dentistry.

## CONCLUSIONS

The current systematic review revealed limited number of studies on EBD, and that none of the studies reported presence of EBD implementation strategies nor evaluation thereof. The dental professionals mainly relied on the electronic resources and person to person communication as the main sources of information for their patient care services.

The study findings indicated a low and below average of knowledge and awareness of EBD and relevant databases for EBD. Poor attitude towards EBD was observed in most primary studies. Practice of EBD and use of scientific evidence databases was low among the dentist in current review. In terms of behavior change, one primary study reported that most of the respondents were aware of EBD concept and that reading a research/scientific article caused them to changetheir practice.

None of the reviewed articles reported implementation of any formal and structured EBD training for clinicians or dental students. A number of barriers that constrained application of EBD were noted in the reviewed studies, which included: lack of interest, the traditional model for decision making is effective and more convenient than EBD, scarcity of reliable and high-level evidence in the world dental literature may require cautious application in our settings, infrastructural limitations (i.e. erratic electricity power supplies, limited access to e-resources, insufficient access to computers and internet facilities), lack of mentors, lack of adequate knowledge and awareness of EBP, limited finance, lack of sufficient time for EBD, and a lack of adequate training opportunities on EBP.

Methodologically, the included studies mainly focused on the cross-sectional survey methods and qualitative interviews. Given the fact that there is low application of EBD and poor use of EBD resources, the study had several implications to improve practice, policy and research methods, as shown below:

Practically, the study recommends the following: Dental schools and hospitals need to build capacity to dental practitioners, faculty and students in the form of continuing educational programs and incorporation of EBD modules into degree/certificate programs. These training programs should inform them about evidence based online databases and resources to increase accuracy of their clinical decision making; Dental schools and hospitals libraries need to have promotion plan that will encourage the application of EBD to dental practitioners, faculty and students by using print and electronics means such as posters, leaflets, online videos on the websites etc.; Dental schools and hospitals need to form evidence-based study clubs between different specialties of healthcare in oral health to encourage adoption and use of EBD; Dental schools and hospitals need to ensure that libraries are kept up-to-date and relevant especially in healthcare as well as in the hospitals; Dental schools and hospitals need to improve the ICT infrastructure to ensure there is adequate access to computers, internet and electrical power for effective application of EBD; Responsible professional Council should give directives tothe dental practitioners to regularly attend courseson EBD and to include it into their daily clinical practice; Methodologically, the study recommends a need for a more rigorous mixed method studies that will combine both quantitative and qualitative studies; The study also recommends the need for allocation of funding for future research on evidence-based dentistry in the region; In terms of policy, dental schools and hospitals need to set up polices and guidelines that will encourage oral health care providers to refer to peer reviewed sources to ensure that they utilize reliable current and evidence-based information for patient management.
